# Stochastic Model for the Internal Transfer Kinetics of Cargo in Carriers with Two Compartments

**DOI:** 10.3390/membranes15120351

**Published:** 2025-11-23

**Authors:** Faruk Hossain, Guilherme Volpe Bossa, Sylvio May

**Affiliations:** 1 Department of Physics, North Dakota State University, Fargo, ND 58108-6050, USA; mdfaruk.hossain.2@ndsu.edu; 2 Institute of Mathematical and Physical Sciences, Universidad Austral de Chile, Valdivia 5090000, Chile; guilherme.vbossa@gmail.com

**Keywords:** stochastic model, two-compartment, Fokker–Planck equation, lipid vesicle, liposome

## Abstract

Lipid vesicles and related nanocarriers often contain two compartments, such as the inner and outer leaflets of a bilayer membrane between which amphipathic molecules can migrate. We develop a stochastic model for describing the transfer kinetics of cargo between the two compartments in an ensemble of carriers, neglecting inter-carrier exchange to focus exclusively on intra-carrier redistribution. Starting from a set of rate equations, we examine the Gaussian regime in the limit of low cargo occupation where Gaussian and Poissonian statistics overlap. We derive a Fokker–Planck equation that we solve analytically for any initial cargo distribution among the carriers. Moments of the predicted distributions and examples, including a comparison between numerical solutions of the rate equations and analytic solutions of the Fokker–Planck equation, are presented and discussed, thereby establishing a theoretical foundation to study coupled intra- and inter-carrier transport processes in mobile nanocarrier systems.

## 1. Introduction

Mobile nanocarriers are used to deliver drug molecules [1], nucleic acids [2], imaging agents [3], proteins and peptides [4], vaccines [5], and even small metabolites or signaling molecules [6,7]. They come in the form of liposomes, polymeric nanoparticles, micelles, dendrimers, lipid nanoparticles, inorganic nanoparticles, and other engineered nanostructures designed for targeted and controlled delivery [1,8]. Release of cargo from nanocarriers can occur passively through diffusion or gradual degradation of the carrier matrix, or actively in response to specific triggers such as pH, redox gradients, temperature, electric and magnetic fields, or light [9].

The passive release of cargo from a nanocarrier can occur through diffusion out of the carrier into the ambient medium or through collisions with other carriers [10]. For example, a liposome loaded with drug molecules can lose its content over time by diffusion of cargo into the aqueous environment or by random transfer of cargo into a different liposome or another target object upon collision. The acquisition of new cargo through the same two transfer modes is equally possible. Detailed modeling of these mechanisms plays an important role in understanding drug release kinetics and optimizing carrier design as well as in formulating release systems that combine passive and stimulus-responsive release [11,12,13].

Liposomes have been studied extensively as carrier vehicles for drug molecules [14], with more than a dozen formulations being currently approved by the U.S. Food and Drug Administration and the European Medicines Agency for clinical use in cancer therapy, antifungal treatment, pain management, and vaccine delivery [15,16]. The association of a drug with a liposome depends on its hydrophobicity profile. Hydrophilic drugs such as doxorubicin [17] or gemcitabine [18] localize in the aqueous core, with the bilayer acting as a barrier, whereas poorly soluble hydrophobic or amphipathic drugs such as amphotericin B [19] or temoporfin [20] embed in the lipid membrane. Even highly hydrophobic drugs show partial polarity [21], leading to asymmetric leaflet interactions, similar to cholesterol with its hydrophobic backbone buried in the bilayer and hydroxyl group anchored at the headgroup region [22]. Thus, bilayer-associated drug molecules are expected to reside predominantly in two states, being associated either with the external or internal leaflet of the membrane.

**Figure 1:**
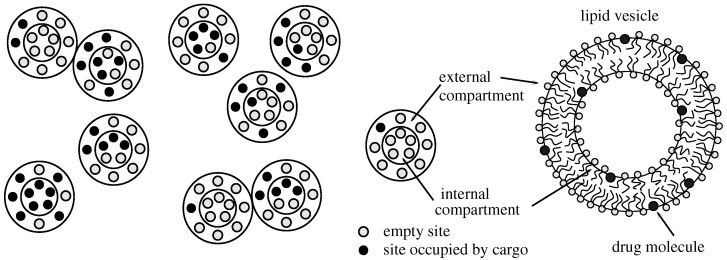
Ensemble of carriers that consist of two compartments, external and internal. Each compartment contains sites that can be empty (gray bullets) or are occupied by a cargo item (black bullets). Upon collisions between carriers, cargo can be transferred from the external compartment of one carrier to the external compartment of another carrier. Cargo can also migrate between the two compartments of a given carrier. In the present work, we study the internal, intra-carrier migration of cargo between the two compartments in the absence of inter-carrier transport. The right side of the figure shows a lipid vesicle as an example of a nanocarrier with amphipathic drug molecules being associated with either the internal or external membrane leaflet.

The present work aims at contributing to the development of a stochastic model for the transfer kinetics of cargo among mobile nanocarriers. The general scope is illustrated in Figure 1, which shows a schematic representation of carriers with two compartments, external and internal. Although not being displayed in Figure 1, different types of carriers may be present in a general system. Each compartment contains identical sites that are either empty or occupied by a single cargo item.

Upon the collision of two carriers, cargo residing in the external compartment of one carrier can migrate to the external compartment of another carrier. Cargo can also migrate between the two compartments of a given carrier. Hence, the migration from the internal compartment of a carrier to the internal compartment of another carrier must proceed via the two external compartments of the two carriers. Modeling the stochastic time-evolution of a suitable distribution function is a challenging task. In a preceding study [23], we have cast the problem into a set of rate equations and identified an analytic solution in the limit that each carrier contains only one single compartment, which is always filled with a sufficiently large number of cargo but is never even close to be filled completely. We have referred to that limit as the Gaussian regime at low occupation. In the present study, we consider the presence of an external and internal compartment in each carrier and allow for the transfer of cargo between the two. However, we shall not allow for the transfer of cargo between different carriers. This restriction to intra-carrier transport is a significant simplification that renders our stochastic model equivalent to first-order unimolecular reactions [24,25]. Yet, in contrast to having only one “reaction chamber”, our system consists of an ensemble of carriers and thus of one “reaction chamber” for each subpopulation of carriers with fixed total cargo content.

Studying only the internal transfer kinetics of cargo in carriers is a necessary step toward solving the composite problem of allowing for transfer both between and within carriers. Yet, even when ignoring transfer between carriers, the remaining two-compartment stochastic model is of interest because, as we show below, it allows us to express the rate equations as a Fokker–Planck equation in exactly the same limit—the Gaussian regime at low occupation—as in our preceding study [23]. We solve the Fokker–Planck equation and analyze its predictions for three specific examples, including a comparison with numerical solutions of the rate equations. Our work thus paves the way to address the full problem: a multicomponent ensemble of two-compartment carriers with both internal and collision-driven inter-carrier cargo exchange, which we plan to investigate in a future study.

We note that our present work applies stochastic modeling to material transport between two adjacent compartments such as the two leaflets of a lipid membrane. Transport processes within cells are frequently modeled stochastically [26], but transport between membrane compartments is most often described using kinetic frameworks. Stochastic approaches remain underexplored in this context, largely due to limited experimental data that would motivate going beyond simple kinetics. Notable exceptions are studies by Shirt-Ediss et al. [27] and by Grosfils and Losada-Pérez [28]. The former develops a stochastic, coarse-grained model of lipid uptake, release, and competitive growth among heterogeneous protocell vesicles, while the latter introduces a direct lipid-transfer model to predict changes in liposome size and composition.

## 2. Theoretical Model and Discussion

We describe the cargo distribution by the quantity $y_{i,n}\left(t\right)$, denoting the number of carriers that, at a given time *t*, contain *i* cargo items in their external compartment and *n* cargo items in their internal compartment. Each carrier possesses two compartments with identical binding sites, $m_{E}$ sites in the external and $m_{I}$ sites in the internal compartment. Every site can either be vacant or host one single cargo molecule. The total number of carriers

(1) $$\begin{array}{c} N=\sum_{i=0}^{m_{E}}\sum_{n=0}^{m_{I}}y_{i,n}\left(t\right) \end{array}$$

is conserved. Cargo can be exchanged between the external and internal compartments of each individual carrier. Hence, the first moments

(2) $$\begin{array}{c} M_{E}\left(t\right)=\sum_{i=0}^{m_{E}}\sum_{n=0}^{m_{I}}i\;y_{i,n}\left(t\right),\;M_{I}\left(t\right)=\sum_{i=0}^{m_{E}}\sum_{n=0}^{m_{I}}n\;y_{i,n}\left(t\right) \end{array}$$

of $y_{i,n}\left(t\right)$ yield time-dependent total numbers of cargo in the external and internal compartment, respectively. The total number $M_{E}\left(t\right)+M_{I}\left(t\right)=M$ of cargo in the external and internal compartments of all carriers is conserved. As discussed in the Introduction, we do not consider transfer of cargo among different carriers, implying that $\sum_{i=0}^{v}\;y_{i,v-i}\left(t\right)$ does not depend on time for any fixed *v* with $0\leq v\leq m_{E}+m_{I}$ and the assumption that $y_{i,n}\left(t\right)=0$ if $i>m_{E}$ or $n>m_{I}$.

### 2.1. Rate Equations

**Figure 2:**
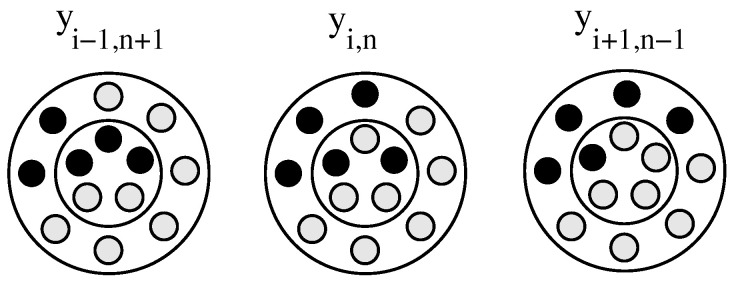
Schematic representation of a carrier with i=3, n=2, $m_{E}=8$, $m_{I}=5$ (**middle**). Inner and outer compartment are separated by the inner circle; empty sites are in gray and filled ones in black. Transfer of a cargo item from the external to the internal compartment results in i=2, n=3 (**left**). Transfer of a cargo item from the internal to the external compartment results in i=4, n=1 (**right**).

To set up rate equations for the kinetics of intra-carrier transfer of cargo, we display, in Figure 2, three carriers that illustrate the processes contributing to the rate of change of $y_{i,n}$.

The population number $y_{i,n}$ increases for a transfer (i−1,n+1)→(i,n) and for a transfer (i+1,n−1)→(i,n). Similarly, the population number $y_{i,n}$ decreases for a transfer (i,n)→(i−1,n+1) and for a transfer (i,n)→(i+1,n−1). We associate each transfer process with a rate constant, $K_{EI}$ for the transfer of cargo from the external to the internal compartment, and $K_{IE}$ for the transfer of cargo from the internal to the external compartment. The rate constants $K_{EI}$ and $K_{IE}$ reflect passive transport properties across an energy barrier that separates the internal and external compartments. For lipid vesicles, the energy barrier originates in the hydrophobic core of the bilayer, which limits the spontaneous flip-flop of hydrophilic or amphiphilic drug molecules between the two leaflets. Permeabilities and flip-flop rates have been studied extensively, both experimentally [29] and via computer simulations [30], for lipid membranes. They are found to depend on a multitude of factors, including steric interactions, hydrogen bond formation, membrane composition and degree of asymmetry, the presence of cholesterol, and pH for ionizable drugs. Note that our use of a fixed, single set of rate constants, $K_{EI}$ and $K_{IE}$, neglects cooperativity of transfer processes [31] and variations due to structural and chemical heterogeneities such as locally different compositions in a multicomponent lipid vesicle [32,33]. In our present model, transfer events are independent from each other. Also note that large differences of $K_{EI}$ and $K_{IE}$ compared to the rate constants associated with inter-carrier transfer will alter the release profile of drug molecules from liposomes away from simple exponential behavior [12]. This indeed was observed for temoporphin upon changing the lipid composition of the host vesicles [34].

The rate of change of $y_{i,n}$ takes the form of a chemical Master equation:

(3) $$\begin{array}{c} \frac{dy_{i,n}\left(t\right)}{dt}=K_{EI}\left[g_{m_{I}}^{i+1\to n-1}y_{i+1,n-1}\left(t\right)-g_{m_{I}}^{i\to n}y_{i,n}\left(t\right)\right]+K_{IE}\left[g_{m_{E}}^{n+1\to i-1}y_{i-1,n+1}\left(t\right)-g_{m_{E}}^{n\to i}y_{i,n}\left(t\right)\right], \end{array}$$

with $y_{-1,n}\left(t\right)=y_{m_{E}+1,n}\left(t\right)=y_{i,-1}\left(t\right)=y_{i,m_{I}+1}\left(t\right)=0$ for all $0\leq i\leq m_{E}$ and $0\leq n\leq m_{I}$. Each term in the rate equations also includes a combinatorial factor [35]:

(4) gmIi→n=mEmE−1i−1mI−1nmEimIn=i1−nmI,gmEn→i=mImI−1n−1mE−1imInmEi=n1−imE,

that accounts for the random statistical occurrence of all possible cargo distributions in a transfer event from the external to the internal compartment (the left of the two equations) and from the internal to the external compartment (the right of the two equations), given the number of cargo in the external and internal compartments is *i* and *n*, respectively. Specifically, the factor $g_{m_{I}}^{i\to n}$ accounts for the number of states mE−1i−1×mI−1n for which a given cargo item can move from an occupied site in the external compartment to an empty site in the internal compartment divided by the total number of available states mEi×mIn, multiplied by the number of available sites in the external compartment. For example, $n=m_{I}$ implies $g_{m_{I}}^{i\to n}=0$, indicating that transfer into a completely filled internal compartment is not possible. Reasoning for the factor $g_{m_{E}}^{n\to i}$ is analogous. Because of the presence of the factors $g_{m_{I}}^{i\to n}$ and $g_{m_{E}}^{n\to i}$, Equation (3) can also be referred to as combinatorial Master equation [36].

Our goal is to determine the distribution $y_{i,n}\left(t\right)$ that solves the Master equation, Equation (3), for a specified initial condition $y_{i,n}\left(t=0\right)$. As shown in Section 2.2 and Section 2.3, this can be achieved analytically under two additional assumptions: applicability of the Gaussian regime and the low-occupation limit. The rate constants $K_{EI}$ and $K_{IE}$, which parameterize the Master equation, are treated as known inputs rather than quantities to be derived. Once $y_{i,n}\left(t\right)$ is obtained, it provides the full time-dependent probability distribution over all carrier populations, from which moments of any order can be computed. A known distribution $y_{i,n}\left(t\right)$ therefore fully characterizes the internal kinetic behavior of the cargo within the carriers.

Based on the rate equations (Equation (3)), we can compute the first moments as defined in Equation (2), resulting in

(5) $$\begin{array}{c} \frac{dM_{E}\left(t\right)}{dt}=-\frac{dM_{I}\left(t\right)}{dt}=K_{IE}M_{I}\left(t\right)-K_{EI}M_{E}\left(t\right)+\left(\frac{K_{EI}}{m_{I}}-\frac{K_{IE}}{m_{E}}\right)\sum_{i=0}^{m_{E}}\sum_{n=0}^{m_{I}}i\;n\;y_{i,n}\left(t\right). \end{array}$$

In the limit $m_{E}\to \infty$ and $m_{I}\to \infty$ or if the condition $K_{EI}m_{E}=K_{IE}m_{I}$ is fulfilled, the final term on the right-hand side of Equation (5) vanishes, and the remaining differential equations for $M_{E}\left(t\right)$ and $M_{I}\left(t\right)$ yield the well-known exponential behavior of a first-order unimolecular reaction [37]:

(6) $$\frac{M_{E}\left(t\right)}{N}=\mu_{E}+e^{-t/\tau }\left(\eta_{E}-\mu_{E}\right),\;\frac{M_{I}\left(t\right)}{N}=\mu_{I}+e^{-t/\tau }\left(\eta_{I}-\mu_{I}\right),$$

with the characteristic time $\tau =1/(K_{EI}+K_{IE})$ and

(7) $$\mu_{E}=\frac{M}{N}\frac{K_{IE}}{K_{EI}+K_{IE}},\;\mu_{I}=\frac{M}{N}\frac{K_{EI}}{K_{EI}+K_{IE}}.$$

Note that $\mu_{E}=M_{E}^{eq}/N$ with $M_{E}^{eq}=M_{E}\left(t\to \infty \right)$ and $\mu_{I}=M_{I}^{eq}/N$ with $M_{I}^{eq}=M_{I}\left(t\to \infty \right)$ denote the number of cargo per carrier in the external and internal compartments, respectively, at equilibrium. Hence, the total amount of cargo per carrier is $\mu_{E}+\mu_{I}=M/N$. Equations (6) satisfy the initial conditions $\eta_{E}=M_{E}\left(t=0\right)/N$ and $\eta_{I}=M_{I}\left(t=0\right)/N$ with $\eta_{E}+\eta_{I}=M/N$. Equations (6) and (7) could be used to obtain $K_{EI}$ and $K_{IE}$ from comparison with experimental data, if such data are available and predict exponential behavior. Equations (6) and (7) are also relevant for the present theoretical work because below, in Section 2.2, we will adopt the Gaussian limit, where $m_{E}\to \infty$ and $m_{I}\to \infty$.

Let us return to the rate equations (Equation (3)). In equilibrium, for t→∞, the function

(8) yi,neq=yi,n(t→∞)=g(i+n)mEipi(1−p)mE−imInqn(1−q)mI−n

takes on a binomial distribution in both *i* and *n* multiplied by a function g(i+n) that depends only on the sum i+n of the two indices and ensures the normalization in Equation (1) is satisfied. The exact form of g(i+n) depends on the initial distribution $y_{i,n}\left(t=0\right)$, as will be discussed below. The probabilities *p* and *q* in Equation (8) relate to the rate constants $K_{EI}$ and $K_{IE}$, the number of sites $m_{E}$ and $m_{I}$, and the overall number of cargo per carrier M/N through the two equations

(9) $$m_{E}\;K_{EI}\;p\left(1-q\right)=m_{I}\;K_{IE}\;\left(1-p\right)q,\;pm_{E}+qm_{I}=\frac{M}{N}.$$

The equation on the left, which is a consequence of detailed balance [36], results from inserting $y_{i,n}^{eq}$ into Equation (3) with $dy_{i,n}/dt=0$, and the equation on the right reflects the conservation of cargo because $M_{E}^{eq}=Npm_{E}$ and $M_{I}^{eq}=Nqm_{I}$. The two relationships in Equation (9) also result from taking the first moments of Equation (8). The probabilities *p* and *q* can be calculated explicitly from Equation (9). The general result is somewhat cumbersome, but if both $m_{E}$ and $m_{I}$ grow large, it simply becomes

(10) $$p=\frac{K_{IE}}{K_{EI}+K_{IE}}\;\frac{M}{m_{E}N},\;q=\frac{K_{EI}}{K_{EI}+K_{IE}}\;\frac{M}{m_{I}N}.$$

That is, Equation (10) becomes valid in the Gaussian limit, where $m_{E}\to \infty$ and $m_{I}\to \infty$. Recalling Equation (7), we observe that $pm_{E}=\mu_{E}$ and $qm_{I}=\mu_{I}$.

### 2.2. Continuum Representation in the Gaussian Limit at Low Occupation

General analytic solutions for the rate equations in Equation (3) are not known to us, not even in the limit of large $m_{E}$ and $m_{I}$. To make progress, we shall consider two approximations. The first is to adopt the *Gaussian limit*, where both $m_{E}$ and $m_{I}$ become large, but such that *p* and *q* adopt values anywhere in the range 0<p<1 and 0<q<1. It is then convenient to adopt a continuum description $y_{i,n}\left(t\right)\to y\left(x,z,t\right)$, where the continuous variables *x* and *z* denote the number of cargo in the external and internal compartments, respectively. That is, the discrete variables *i* and *n* in $y_{i,n}\left(t\right)$ are replaced by the continuous variables *x* and *z* in y(x,z,t).

The moments in Equations (1) and (2) then read $N=\int_{-\infty }^{\infty }dx\int_{-\infty }^{\infty }dz\;y\left(x,z,t\right)$, $M_{E}\left(t\right)=\int_{-\infty }^{\infty }dx\int_{-\infty }^{\infty }dz\;x\;y\left(x,z,t\right)$, and $M_{I}\left(t\right)=\int_{-\infty }^{\infty }dx\int_{-\infty }^{\infty }dz\;z\;y\left(x,z,t\right)$. The binomial distribution in Equation (8), which is adopted in equilibrium, turns into a Gaussian:

(11) $$\begin{array}{c} y^{eq}\left(x,z\right)=g\left(x+z\right)\;\frac{e^{-\frac{{\left(x-m_{E}p\right)}^{2}}{2m_{E}p\left(1-p\right)}}}{\sqrt{2\pi m_{E}p\left(1-p\right)}}\;\frac{e^{-\frac{{\left(z-m_{I}q\right)}^{2}}{2m_{I}q\left(1-q\right)}}}{\sqrt{2\pi m_{I}q\left(1-q\right)}}. \end{array}$$

Instead of the discrete variable i+n in Equation (8), we employ the corresponding continuous variable x+z as argument of the function g(x+z). It is convenient to re-express the function

(12) $$g\left(x+z\right)=\frac{1}{2}f\left(x+z\right)\frac{\sqrt{2\pi \left[m_{E}p\left(1-p\right)+m_{I}q\left(1-q\right)\right]}}{e^{-\frac{{\left(x-m_{E}p+z-m_{I}q\right)}^{2}}{2\left[m_{E}p\left(1-p\right)+m_{I}q\left(1-q\right)\right]}}}$$

in terms of a function f(x+z). Equation (12) can be viewed as the definition of the new function f(x+z), given g(x+z) is known. The advantage of using f(x+z) is the simple normalization

(13) $$\frac{1}{2}\int_{-\infty }^{\infty }dv\;f\left(v\right)=N$$

that this function must fulfill in order to ensure normalization of $y_{i,n}\left(t\right)$ according to Equation (1) is satisfied.

In the Gaussian limit, Equation (3) can be subjected to a series expansion, known as Kramers–Moyal expansion [36], of i+1→x+Δx and n+1→z+Δz in terms of Δx and Δz, followed by setting Δx=Δz=1. Up to first order, the result of the expansion is

(14) $$\frac{dy}{dt}=\left(\frac{\partial }{\partial x}-\frac{\partial }{\partial z}\right)\left[-K_{IE}\left(1-\frac{x}{m_{E}}\right)zy+K_{EI}\left(1-\frac{z}{m_{I}}\right)xy\right].$$

Being of first order, this equation contains drift but no fluctuation terms. Solutions of Equation (14) would describe how initial delta-peaks would move in time without widening due to fluctuations. Fluctuations would enter Equation (14) as terms associated with second-order derivatives. These terms would emerge from extending the first-order Kramers–Moyal expansion that leads from Equation (3) to Equation (14) to a second-order expansion, followed by a linearization of the coefficients associated with the drift and fluctuation terms to model an Ornstein–Uhlenbeck process [36]. A simpler way forward to identify the fluctuation terms is to adopt a second approximation, the *low occupation limit*, where the number of occupied sites in the external and internal compartment is always much smaller than the number of available sites. The corresponding conditions $M\ll m_{E}N$ and $M\ll m_{I}N$ imply p≪1 and q≪1. Note that $\mu_{E}=pm_{E}$ and $\mu_{I}=qm_{I}$ both remain finite. Mathematically, the small occupation limit uses the Gaussian distribution $e^{-{\left(x-\mu \right)}^{2}/\left(2\mu \right)}/\sqrt{2\pi \mu }$ to approximate a Poisson distribution $e^{-\mu }\mu^{x}/x!$ for large mean value μ [38]. The equilibrium distribution specified in Equations (11) and (12) then reads

(15) $$y^{eq}\left(x,z\right)=\frac{1}{2}\;f\left(x+z\right)\;\frac{\sqrt{2\pi (\mu_{E}+\mu_{I})}}{e^{-\frac{{\left(x-\mu_{E}+z-\mu_{I}\right)}^{2}}{2(\mu_{E}+\mu_{I})}}}\;\frac{e^{-\frac{{\left(x-\mu_{E}\right)}^{2}}{2\mu_{E}}}}{\sqrt{2\pi \mu_{E}}}\;\frac{e^{-\frac{{\left(z-\mu_{I}\right)}^{2}}{2\mu_{I}}}}{\sqrt{2\pi \mu_{I}}},$$

with the function f(x+z) satisfying the normalization in Equation (13). In the low occupation limit, the terms $x/m_{E}$ and $z/m_{I}$ in Equation (14) are negligibly small, and it becomes straightforward to identify the missing second-order fluctuation term by comparing the stationary solutions of Equation (14) with Equation (15). The resulting equation

(16) $$\tau \;\frac{dy}{dt}=\left(\frac{\partial }{\partial x}-\frac{\partial }{\partial z}\right)\left[\frac{\mu_{I}x-\mu_{E}z}{\mu_{E}+\mu_{I}}y+\frac{\mu_{E}\mu_{I}}{\mu_{E}+\mu_{I}}\left(\frac{\partial y}{\partial x}-\frac{\partial y}{\partial z}\right)\right]$$

is a Fokker–Planck equation [36] for the distribution y(x,z,t), with $\tau =1/(K_{EI}+K_{IE})$. We reiterate that the drift term, which is associated with the first derivatives, follows from Equation (14) in the limit of large $m_{E}$ and $m_{I}$ and employs the definitions of $\mu_{E}$ and $\mu_{I}$ in Equation (7). The fluctuation term, which accounts for the second-order derivatives in Equation (16), ensures the equilibrium distribution $y^{eq}\left(x,z\right)=y\left(x,z,t\to \infty \right)$ of Equation (16) is given by Equation (15).

The two assumptions on which Equation (16) relies, the Gaussian regime and the low-occupation limit, are both expected to hold for lipid vesicles used as drug carriers. A vesicle of 100 nm diameter contains on the order of $10^{5}$ lipids. Even if as many as $10^{2}$ lipids would be needed to form one independent site for a drug molecule, the resulting $10^{3}$ sites would allow hundreds of drug molecules to be loaded without violating the assumptions underlying Equation (16). At least on the order of $10^{1}$, drug molecules should be present in each leaflet at any given time to ensure the Gaussian regime would not have to be replaced by the Poisson regime.

### 2.3. Solution of the Fokker–Planck Equation

In order to solve Equation (16), we introduce the new independent variables u=x−z and v=x+z. This is motivated by the conservation of the total number of cargo, *v*, in each carrier population y((v+u)/2,(v−u)/2,t) as *u* varies. The function $\tilde{y}\left(u,v,t\right)=y\left(\left(v+u\right)/2,\left(v-u\right)/2,t\right)$ then satisfies the equation

(17) $$\tau \frac{d\tilde{y}}{dt}=\frac{\partial }{\partial u}\left\{\left[u-v\frac{\mu_{E}-\mu_{I}}{\mu_{E}+\mu_{I}}\right]\tilde{y}+\frac{4\mu_{E}\mu_{I}}{\mu_{E}+\mu_{I}}\frac{\partial \tilde{y}}{\partial u}\right\}.$$

Because we adopted the Gaussian limit, the new variables *u* and *v* vary from −∞ to *∞*. Also, note that dxdz=dudv/2. Hence, solutions of Equation (17) must conserve the number of carriers $N=1/2\times \int_{-\infty }^{\infty }dv\int_{-\infty }^{\infty }du\;\tilde{y}\left(u,v,t\right)$ and the total number of cargo $M=1/2\times \int_{-\infty }^{\infty }dv\;v\;\int_{-\infty }^{\infty }du\;\tilde{y}\left(u,v,t\right)$. Let us specify an initial distribution $\tilde{y}\left(u,v,t=0\right)={\tilde{y}}_{0}\left(u,v\right)$. Solutions of Equation (17):

(18) $$\tilde{y}\left(u,v,t\right)=\int_{-\infty }^{\infty }du^{\prime }G\left(u,v,t|u^{\prime }\right)\;{\tilde{y}}_{0}\left(u^{\prime },v\right),$$

can be constructed using the Green’s function:

(19) $$G\left(u,v,t|u^{\prime }\right)=\frac{1}{\sqrt{2\pi \sigma (t)}}\;e^{-\frac{{\left[u-\mu (v,t|u^{\prime })\right]}^{2}}{2\sigma (t)}},$$

with

(20) $$\begin{array}{c} \mu \left(v,t|u^{\prime }\right)=u^{\prime }e^{-t/\tau }+v\frac{\mu_{E}-\mu_{I}}{\mu_{E}+\mu_{I}}\left(1-e^{-t/\tau }\right),\;\sigma \left(t\right)=\frac{4\mu_{E}\mu_{I}}{\mu_{E}+\mu_{I}}\left(1-e^{-2t/\tau }\right). \end{array}$$

The Green’s function $G(u,v,t|u^{\prime })$, defined through Equations (19) and (20), satisfies the Fokker–Planck equation (Equation (17)) and is normalized such that $\int_{-\infty }^{\infty }duG\left(u,v,t|u^{\prime }\right)=1$. It initially produces a delta-peak at position $u^{\prime }$—that is, the Green’s function satisfies the initial condition $G\left(u,v,t\to 0|u^{\prime }\right)=\delta \left(u-u^{\prime }\right)$, where δ(u) denotes the Dirac delta function. Hence, Equation (18) reproduces the initial distribution ${\tilde{y}}_{0}\left(u,v\right)$ in the limit t→0. In the opposite limit, at t→∞, the Green’s function

(21) $$G\left(u,v,t\to \infty |u^{\prime }\right)=\frac{1}{\sqrt{2\pi \frac{4\mu_{E}\mu_{I}}{\mu_{I}+\mu_{E}}}}\;e^{-\frac{1}{2}\;\frac{{\left(u-v\frac{\mu_{E}-\mu_{I}}{\mu_{I}+\mu_{E}}\right)}^{2}}{\frac{4\mu_{E}\mu_{I}}{\mu_{I}+\mu_{E}}}}$$

is independent of $u^{\prime }$, and Equation (18) thus yields

(22) $$\tilde{y}\left(u,v,t\to \infty \right)=G\left(u,v,t\to \infty |u^{\prime }\right)\;\int_{-\infty }^{\infty }du^{\prime }{\tilde{y}}_{0}\left(u^{\prime },v\right).$$

The normalization $N=1/2\times \int_{-\infty }^{\infty }dv\int_{-\infty }^{\infty }du\;\tilde{y}\left(u,v,t\right)$ together with Equation (13) implies $f\left(v\right)=\int_{-\infty }^{\infty }du\;\tilde{y}\left(u,v,t\right)$ is conserved. The integral in Equation (22) is thus $\int_{-\infty }^{\infty }du^{\prime }\;{\tilde{y}}_{0}\left(u^{\prime },v\right)=f\left(v\right)$. Using this, together with Equation (21) and the definitions u=x−z and v=x+z, renders Equation (22) identical to Equation (15), thus reproducing the correct equilibrium distribution. While the equilibrium distribution is Gaussian, the non-equilibrium distributions need not be Gaussians.

It is also interesting to calculate moments of our solution $\tilde{y}\left(u,v,t\right)$ in Equation (18). The first moment with respect to *v* yields

(23) $$\begin{array}{ccc} \langle v\rangle & = & \frac{1}{2N}\int_{-\infty }^{\infty }du\int_{-\infty }^{\infty }dv\;v\;\tilde{y}\left(u,v,t\right)=\frac{1}{2N}\int_{-\infty }^{\infty }du\int_{-\infty }^{\infty }dv\;v\;\int_{-\infty }^{\infty }du^{\prime }G\left(u,v,t|u^{\prime }\right)\;{\tilde{y}}_{0}\left(u^{\prime },v\right)\\ & = & \frac{1}{2N}\int_{-\infty }^{\infty }du^{\prime }\int_{-\infty }^{\infty }dv\;v\;{\tilde{y}}_{0}\left(u^{\prime },v\right)=\frac{M}{N}, \end{array}$$

demonstrating that our solution $\tilde{y}\left(u,v,t\right)$ indeed conserves *M*. It can analogously be concluded that higher moments in *v* are all conserved, implying that distribution functions y(x,z,t) can change with time in the x−z direction but not in the x+z direction. The first moment with respect to *u* results in

(24) $$\begin{array}{ccc} \langle u\rangle & = & \frac{1}{2N}\int_{-\infty }^{\infty }du\int_{-\infty }^{\infty }dv\;u\;\tilde{y}\left(u,v,t\right)=\frac{1}{2N}\int_{-\infty }^{\infty }du\int_{-\infty }^{\infty }dv\;u\;\int_{-\infty }^{\infty }du^{\prime }G\left(u,v,t|u^{\prime }\right)\;{\tilde{y}}_{0}\left(u^{\prime },v\right)\\ & = & \mu_{E}-\mu_{I}+e^{-t/\tau }\left(\eta_{E}-\eta_{I}-\mu_{E}+\mu_{I}\right)=\frac{M_{E}\left(t\right)}{N}-\frac{M_{I}\left(t\right)}{N}, \end{array}$$

thus reproducing $M_{E}\left(t\right)$ and $M_{I}\left(t\right)$ according to Equation (6). To transition from the first to the second line of Equation (24), we have made use of $\mu_{E}+\mu_{I}=M/N$ (see Equation (7)), $\tau =1/(K_{IE}+K_{EI})$, $\eta_{E}=M_{E}\left(0\right)/N$, $\eta_{I}=M_{I}\left(0\right)/N$, and $\int_{-\infty }^{\infty }du\;u\;G\left(u,v,t|u^{\prime }\right)=\mu \left(v,t|u^{\prime }\right)$ (see Equation (20)). Calculation of the second moment with respect to *u* gives rise to

(25) $$\begin{array}{ccc} \langle u^{2}\rangle =\frac{1}{2N}\int_{-\infty }^{\infty }du\int_{-\infty }^{\infty }dv\;u^{2}\;\tilde{y}\left(u,v,t\right) & = & \frac{4\mu_{E}\mu_{I}}{\mu_{E}+\mu_{I}}\left(1-e^{-2t/\tau }\right)+e^{-2t/\tau }{\langle u^{2}\rangle }_{0}\\ & + & 2\frac{\mu_{E}-\mu_{I}}{\mu_{E}+\mu_{I}}\left(e^{-t/\tau }-e^{-2t/\tau }\right){\langle uv\rangle }_{0}\\ & + & {\left(\frac{\mu_{E}-\mu_{I}}{\mu_{E}+\mu_{I}}\right)}^{2}{\left(1-e^{-t/\tau }\right)}^{2}{\langle v^{2}\rangle }_{0}, \end{array}$$

where we have used $\int_{-\infty }^{\infty }du\;u^{2}\;G\left(u,v,t|u^{\prime }\right)=\mu {\left(v,t|u^{\prime }\right)}^{2}+\sigma \left(t\right)$, and where we have defined the initial value (at t=0) of the second moment:

(26) $$\begin{array}{c} {\langle u^{2}\rangle }_{0}=\frac{1}{2N}\int_{-\infty }^{\infty }du\int_{-\infty }^{\infty }dv\;u^{2}\;{\tilde{y}}_{0}\left(u,v\right), \end{array}$$

with analogous definitions for ${\langle uv\rangle }_{0}$ and ${\langle v^{2}\rangle }_{0}$. Note $\langle v^{2}\rangle ={\langle v^{2}\rangle }_{0}$ is conserved, as discussed above. Equation (25) demonstrates how the second moment with respect to *u* propagates in time from the initial ${\langle u^{2}\rangle }_{0}$ to the final value:

(27) $$\begin{array}{c} {\langle u^{2}\rangle }_{eq}=\frac{1}{2N}\int_{-\infty }^{\infty }du\int_{-\infty }^{\infty }dv\;u^{2}\;{\tilde{y}}^{eq}\left(u,v\right)=\frac{4\mu_{E}\mu_{I}}{\mu_{E}+\mu_{I}}+{\left(\frac{\mu_{E}-\mu_{I}}{\mu_{E}+\mu_{I}}\right)}^{2}{\langle v^{2}\rangle }_{0}, \end{array}$$

which depends on ${\langle v^{2}\rangle }_{0}$. That is, if two initial distributions $y^{eq}\left(x,z\right)$ differ in their second moment ${\langle v^{2}\rangle }_{0}$, then their equilibrium distributions will also be different, given that $\mu_{E}\neq \mu_{I}$. Based on $\langle u^{2}\rangle$ in Equation (25) and 〈u〉 in Equation (24), the calculation of the variance $\langle {\left(u-\langle u\rangle \right)}^{2}\rangle =\langle u^{2}\rangle -{\langle u\rangle }^{2}$ is straightforward. As pointed out, the corresponding variance in the *v*-direction, $\langle {\left(v-\langle v\rangle \right)}^{2}\rangle =\langle v^{2}\rangle -{\langle v\rangle }^{2}={\langle v^{2}\rangle }_{0}-{\left(M/N\right)}^{2}$ is independent of time. We note that any higher moment of the solution $\tilde{y}\left(u,v,t\right)$ in Equation (18) can also be calculated analytically.

### 2.4. Three Specific Examples

The following three examples illustrate simple cases for the calculation of y(x,z,t) and corresponding moments from a given initial distribution $y_{0}\left(x,z\right)$, rate constants $K_{EI}$ and $K_{IE}$, and overall cargo-to-carrier ratio $M/N=\mu_{E}+\mu_{I}$. The examples are selected to facilitate understanding of how distributions y(x,z,t) propagate in the x,z-plane. Because of the specific scope of the present stochastic model as one component of an integrated two-component modeling effort, we do not attempt to identify the matching of our current model predictions with experimental observations.

#### 2.4.1. First Example

Assume all *N* carriers contain exactly the same number of cargo, M/N, in each carrier, and all of that is initially contained exclusively in the external compartment, $M_{E}\left(0\right)=M$ and $M_{I}\left(0\right)=0$. Our assumptions amount to the initial distribution function ${\tilde{y}}_{0}\left(u,v\right)=2N\;\delta \left(u-M/N\right)\;\delta \left(v-M/N\right)$. The general solution in Equation (18) then reads $\tilde{y}\left(u,v,t\right)=2NG\left(u,v,t|v\right)\delta \left(v-M/N\right)$. Because of the presence of the delta-peak δ(v−M/N), it is convenient and sufficient to focus on

(28) $$\begin{array}{c} \int_{-\infty }^{\infty }dv\;\tilde{y}\left(u,v,t\right)=2N\;G\left(u,\frac{M}{N},t|\frac{M}{N}\right)=\frac{2N}{\sqrt{2\pi \sigma (t)}}\;e^{-\frac{{\left[u-\mu_{E}+\mu_{I}\left(1-2e^{-t/\tau }\right)\right]}^{2}}{2\sigma (t)}}, \end{array}$$

with σ(t) specified in Equation (20). Equation (28) describes how the distribution shifts from the initial delta-peak at u=M/N and v=M/N to a Gaussian at $u=\mu_{E}-\mu_{I}$ and v=M/N. Expressed in terms of *x* and *z*, this shift occurs from the initial x=M/N and z=0 to the final $x=\mu_{E}=M_{E}^{eq}/N$ and $z=\mu_{I}=M_{I}^{eq}/N$. Calculation of relevant moments gives rise to 〈v〉=M/N, $\langle {\left(v-\langle v\rangle \right)}^{2}\rangle =0$, $\langle u\rangle =\mu_{E}-\mu_{I}\left(1-2e^{-t/\tau }\right)$, and $\langle {\left(u-\langle u\rangle \right)}^{2}\rangle =\sigma \left(t\right)$. The latter reflects the time evolution of the Green’s function, consistent with our initial distribution ${\tilde{y}}_{0}\left(u,v\right)$ consisting of a delta-peak.

#### 2.4.2. Second Example

We assume an initially Gaussian distribution

(29) $$\begin{array}{c} y_{0}\left(x,z\right)=\frac{N}{2\pi \sqrt{\eta_{E}\eta_{I}}}\;e^{-\frac{{\left(x-\eta_{E}\right)}^{2}}{2\eta_{E}}}e^{-\frac{{\left(z-\eta_{I}\right)}^{2}}{2\eta_{I}}}, \end{array}$$

where we recall from Equation (6) the initial numbers of cargo per carrier, $\eta_{E}=M_{E}\left(t=0\right)/N$ and $\eta_{I}=M_{I}\left(t=0\right)/N$ in the two compartments, with $\eta_{E}+\eta_{I}=M/N$ being the combined number. Using ${\tilde{y}}_{0}\left(u,v\right)=y_{0}\left(\left(v+u\right)/2,\left(v-u\right)/2\right)$, we can calculate the function

(30) $$\begin{array}{c} f\left(v\right)=\int_{-\infty }^{\infty }du{\tilde{y}}_{0}\left(u,v\right)=\frac{2N}{\sqrt{2\pi M/N}}e^{-\frac{{\left(v-M/N\right)}^{2}}{2M/N}}. \end{array}$$

Inserting f(v)=f(x+y) into Equation (15) yields the equilibrium distribution

(31) $$\begin{array}{c} y^{eq}\left(x,z\right)=\frac{N}{2\pi \sqrt{\mu_{E}\mu_{I}}}\;e^{-\frac{{\left(x-\mu_{E}\right)}^{2}}{2\mu_{E}}}e^{-\frac{{\left(z-\mu_{I}\right)}^{2}}{2\mu_{I}}}. \end{array}$$

**Figure 3:**
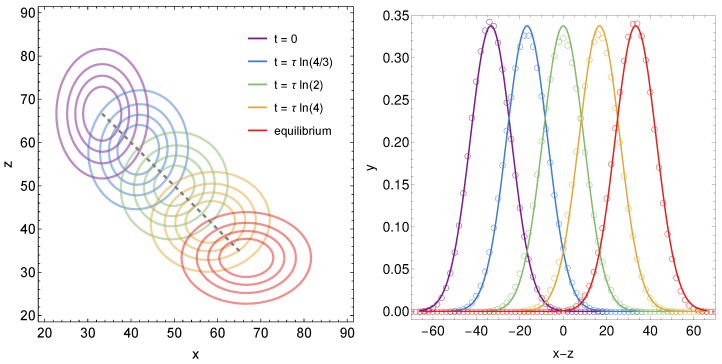
**Left** diagram: The distribution y(x,z,t) according to Equation (32) for M/N=100 as function of *x* and *z* for different times as indicated in the legend. Recall that Equation (32) employs the specific choice $\mu_{E}=\eta_{I}=2/3\times M/N$ and $\mu_{I}=\eta_{E}=1/3\times M/N$. For each time, y(x,z,t) is represented by four contour lines; $y_{0}\left(x,z\right)$ is shown in purple (marked t=0 in the legend) and $y^{eq}\left(x,z\right)$ in red (marked “equilibrium” in the legend). The dashed line specifies the linear path $z_{max}=M/N-x_{max}$ along which the maximum of y(x,z,t) moves. Here, $x_{max}=M_{E}\left(t\right)/N$ and $z_{max}=M_{I}\left(t\right)/N$ are mean values according to Equation (34). **Right** diagram: Cross sections of y(x,z,t) along the dashed line in left diagram, thus fixing x+z=v=M/N=100. That is, y((100+x−z)/2,(100−x+z)/2,t) is plotted for different times *t*, with the same color code as in the legend of the left diagram. The color-matching circles mark numerical solutions of the corresponding discrete rate equations specified in Equation (3) with $K_{EI}=1/\left(3\tau \right)$, $K_{IE}=2/\left(3\tau \right)$, N=100, M=10,000, and $m_{E}=m_{I}=2500$.

Calculating the full kinetic behavior y(x,z,t) from $\tilde{y}\left(u,v,t\right)$ according to Equations (17)–(20) leads to somewhat cumbersome expressions but can, of course, be carried out numerically. Here, we focus on the specific case $\mu_{E}=\eta_{I}=2/3\times M/N$ and $\mu_{I}=\eta_{E}=1/3\times M/N$, where the initial distribution of the cargo in the two compartments is opposite of the preferred one. This leads to the simple analytic result:

(32) $$\begin{array}{ccc} y(x,z,t) & = & y^{eq}\left(x,z\right)e^{-\frac{N}{4M}\left(x+z\right)\left[e^{-2t/\tau }\left(x+z\right)+2e^{-t/\tau }\left(x-2z\right)\right]}, \end{array}$$

where the limiting values for t=0 and t→∞:

(33) $$\begin{array}{ccc} y_{0}\left(x,z\right) & = & \frac{N}{2\pi \sqrt{\frac{M}{3N}\;\frac{2M}{3N}}}\;e^{-\frac{3}{2}\frac{N}{M}{\left(x-\frac{1}{3}\frac{M}{N}\right)}^{2}}\;e^{-\frac{3}{4}\frac{N}{M}{\left(z-\frac{2}{3}\frac{M}{N}\right)}^{2}},\\ y^{eq}\left(x,z\right) & = & \frac{N}{2\pi \sqrt{\frac{M}{3N}\;\frac{2M}{3N}}}\;e^{-\frac{3}{4}\frac{N}{M}{\left(x-\frac{2}{3}\frac{M}{N}\right)}^{2}}\;e^{-\frac{3}{2}\frac{N}{M}{\left(z-\frac{1}{3}\frac{M}{N}\right)}^{2}}, \end{array}$$

agree with Equations (29) and (31). The left diagram of Figure 3 shows a contour plot of y(x,z,t) according to Equation (32) for M/N=100 at the five different times specified in the legend.

The dashed line displays the path

(34) $$\begin{array}{c} \frac{M_{E}\left(t\right)}{N}=\frac{M}{3N}\left(2-e^{-t/\tau }\right),\;\frac{M_{I}\left(t\right)}{N}=\frac{M}{3N}\left(1+e^{-t/\tau }\right), \end{array}$$

along which the maximum of the Gaussian distribution y(x,z,t) moves over time until reaching equilibrium. Equation (34) is identical to Equation (6), with the specific choices $\mu_{E}=2\mu_{I}=2\eta_{E}=\eta_{I}=2M/\left(3N\right)$ of our current example. Because of $M_{E}\left(t\right)+M_{I}\left(t\right)=M$, this path is linear, with a slope −1 of the dashed line in Figure 3. The probability distributions along all lines of slope −1 correspond to a total number of cargo x+z. That is, only u=x−z but not v=x+z changes along a line of slope −1. Note also $M_{E}\left(t\right)=M_{I}\left(t\right)$ in Equation (34) occurs after a time t=τln2. Hence, for the green curves in Figure 3, the cargo is distributed equally among external and internal compartment, $M_{E}/M=M_{I}/M=1/2$. The sequence of the five selected times t/τ=0, ln(4/3), ln(2), ln(4), and *∞*, in Figure 3, corresponds to $M_{E}/M=4/12$, 5/12, 6/12, 7/12, and 8/12.

Moments for our specific example can be calculated using the general formalism in Equations (23)–(25) or, equivalently, directly from the solution in Equation (32), yielding for the variances

(35) $$\langle {\left(v-\langle v\rangle \right)}^{2}\rangle =\frac{M}{N},\;\langle {\left(u-\langle u\rangle \right)}^{2}\rangle =\frac{M}{N}\left[1-\frac{4}{9}\left(e^{-t/\tau }-e^{-2t/\tau }\right)\right].$$

As pointed out above, averaging over *v* yields time-independent results. The corresponding variance in the *u*-direction is M/N for t=0 and t→∞, but adopts a minimum 8/9×M/N at t/τ=ln2. Hence, the variances for the distributions at t=0 (purple) and t→∞ (red) in the left diagram of Figure 3 are the same in the x−z-direction and in the x+z-direction. At all intermediate times, the variance in the x−z-direction is smaller than that in the x+z-direction, with the largest difference for t=τln2 (shown in green).

The right diagram in Figure 3 shows cross-sections of y(x,z,t) along the direction of the dashed line in the left diagram, where x+z=M/N=100 is fixed. Curves are displayed for the same set of times as in the left diagram, with the same color code. The solid lines correspond to y(x,z,t) in Equation (32). The open circles show numerical solutions of the corresponding discrete rate equations, Equation (3), with $K_{EI}=1/\left(3\tau \right)$ and $K_{IE}=2/\left(3\tau \right)$, N=100, M=10,000, and an initial distribution yi,n(t=0)=NmEip0i(1−p0)mE−imInq0n(1−q0)mI−n, where $p_{0}=M/\left(3Nm_{E}\right)$ and $q_{0}=2M/\left(3Nm_{I}\right)$. Optimal agreement is expected in the limit $m_{E}\to \infty$ and $m_{I}\to \infty$. Our calculations were performed for $m_{E}=m_{I}=2500$, which also leads to excellent matching. The comparison in the right diagram of Figure 3 of corresponding numerical solutions for the discrete rate equations in Equation (3) and analytic solutions of the Fokker–Planck equation in Equation (16) reinforces the equivalence of the two approaches.

#### 2.4.3. Third Example

Our final example illustrates the addition of another Gaussian distribution to the initial distribution $y_{0}\left(x,z\right)$. That is, we replace the single Gaussian distribution in Equation (29) by the sum of two Gaussians:

(36) $$\begin{array}{c} y_{0}\left(x,z\right)=\frac{\alpha N}{2\pi \sqrt{\eta_{E}^{\left(1\right)}\eta_{I}^{\left(1\right)}}}\;e^{-\frac{{\left(x-\eta_{E}^{\left(1\right)}\right)}^{2}}{2\eta_{E}^{\left(1\right)}}}\;e^{-\frac{{\left(z-\eta_{I}^{\left(1\right)}\right)}^{2}}{2\eta_{I}^{\left(1\right)}}}+\frac{(1-\alpha )N}{2\pi \sqrt{\eta_{E}^{\left(2\right)}\eta_{I}^{\left(2\right)}}}\;e^{-\frac{{\left(x-\eta_{E}^{\left(2\right)}\right)}^{2}}{2\eta_{E}^{\left(2\right)}}}\;e^{-\frac{{\left(z-\eta_{I}^{\left(2\right)}\right)}^{2}}{2\eta_{I}^{\left(2\right)}}}. \end{array}$$

**Figure 4:**
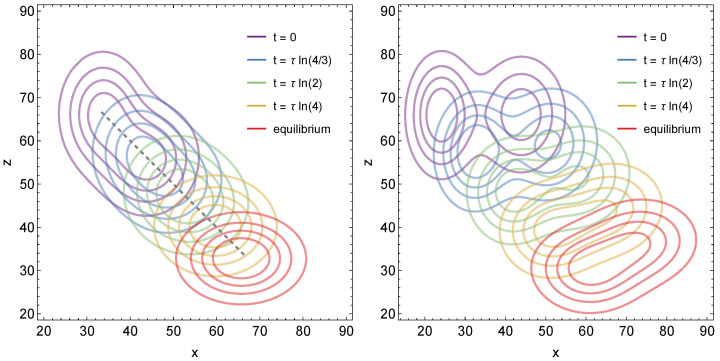
The distribution y(x,z,t) for $\mu_{E}=2/3\times M/N$ and $\mu_{I}=1/3\times M/N$ with M/N=100 as function of *x* and *z* for different times as indicated in the legend. The initial distribution $y_{0}\left(x,z\right)$ is the sum of two Gaussians according to Equation (36) with α=1/2. Our parameter choices for the **left** diagram, $\eta_{E}^{\left(1\right)}=1/3\times M/N$, $\eta_{I}^{\left(1\right)}=2/3\times M/N$, $\eta_{E}^{\left(2\right)}/\eta_{E}^{\left(1\right)}=1+e^{-4/10}$, and $\eta_{I}^{\left(2\right)}/\eta_{I}^{\left(1\right)}=1-e^{-4/10}$, represent a redistribution of the initial distribution $y_{0}\left(x,z\right)$ in Figure 3 along lines of fixed x+z=v, thus ensuring that the equilibrium distributions $y^{eq}\left(x,z\right)$ are the same. In contrast, the redistribution in the **right** diagram, enforced through our parameter choice $\eta_{E}^{\left(1\right)}=\left(1/3-1/10\right)\times M/N$, $\eta_{E}^{\left(2\right)}=\left(1/3+1/10\right)\times M/N$, $\eta_{I}^{\left(1\right)}=\eta_{I}^{\left(2\right)}=2/3\times M/N$, is along the *x*-axis, resulting in a different equilibrium distribution $y^{eq}\left(x,z\right)$.

The two diagrams of Figure 4 show y(x,z,t) for two specific choices for the parameters $\eta_{E}^{\left(1\right)}$, $\eta_{E}^{\left(2\right)}$, $\eta_{I}^{\left(1\right)}$, $\eta_{I}^{\left(2\right)}$, and α. The rate constants $K_{EI}$ and $K_{IE}$ as well as M/N are the same as in Figure 3.

In the left diagram, the two Gaussians of Figure 3 for t=0 and t/τ=0.4 are added, with α=1/2 giving each contribution the same weight. That is, $\eta_{E}^{\left(1\right)}=1/3\times M/N$, $\eta_{I}^{\left(1\right)}=2/3\times M/N$, $\eta_{E}^{\left(2\right)}/\eta_{E}^{\left(1\right)}=1+e^{-4/10}$, and $\eta_{I}^{\left(2\right)}/\eta_{I}^{\left(1\right)}=1-e^{-4/10}$ were specified. Contour lines of the resulting initial distribution $y_{0}\left(x,z\right)$ are displayed in purple. The cumulative probabilities $f\left(x+z\right)=f\left(v\right)=\int_{-\infty }^{\infty }duy_{0}\left(u,v\right)$ as well as $\mu_{E}$ and $\mu_{I}$ are the same in Figure 3 and the left diagram of Figure 4. Hence, the equilibrium distributions $y^{eq}\left(x,z\right)$ (with contour lines shown in red) are the same.

In the right diagram, we separate the two Gaussians along the *x*-axis by choosing $\eta_{E}^{\left(1\right)}=\left(1/3-1/10\right)\times M/N$, $\eta_{E}^{\left(2\right)}=\left(1/3+1/10\right)\times M/N$, $\eta_{I}^{\left(1\right)}=\eta_{I}^{\left(2\right)}=2/3\times M/N$ as well as α=1/2. The purple contour lines represent $y_{0}\left(x,z\right)$. Here, the initial distribution evolves toward a different final distribution (with contour lines shown in red) because f(x+z) for the right diagram of Figure 4 is different from f(x+z) for Figure 3 and the left diagram of Figure 4.

## 3. Conclusions

This work presents a stochastic model for the kinetics of cargo transfer among two compartments that each contain identical sites but different affinities. We show that rate equations give rise to a Fokker–Planck equation in the continuum and low occupation limits, where the Gaussian and Poisson regimes of a binomial distribution overlap. That implies a large number of cargo must be present yet without ever approaching full occupation of either compartment. Analytic solutions of the Fokker–Planck equation are presented by identifying a Green’s function and discussed in terms of moments and three specific examples.

We emphasize that our model represents a step towards rationalizing the kinetics of collision-dominated cargo transfer among mobile two-compartment nanocarriers such as drug molecules associated with the inner or outer leaflets of lipid vesicles. While a preceding study has focused on the inter-carrier transfer of cargo [23], the present one addresses the intra-carrier transport. Combining the two models into a single comprehensive framework is non-trivial because each carrier type generates its own Fokker–Planck equation with implicitly time-dependent coefficients. These coefficients arise from the eigenvalues and eigenvectors of a rate matrix that is itself influenced by the internal transfer kinetics analyzed in this work. Even so, future work will explore if an analytic treatment of the combined problem can be achieved. An exact solution of the combined model, including both intra-carrier and inter-carrier transfer, would provide a more general framework to discuss the transport of cargo through mobile multicomponent nanocarriers as present in pharmaceutical and technological applications including drug delivery, food science, dry powder coating, and host–guest complexes.

## Data Availability

The original contributions presented in this study are included in the article. Further inquiries can be directed to the corresponding author.
